# Temporal trends in opportunistic citizen science reports across multiple taxa

**DOI:** 10.1007/s13280-021-01550-w

**Published:** 2021-03-29

**Authors:** Jonas Knape, Stephen James Coulson, René van der Wal, Debora Arlt

**Affiliations:** 1 Department of Ecology, Swedish University of Agricultural Sciences, Inst för Ekologi, Box 7044, 75007 Uppsala, Sweden; 2 Swedish Species Information Centre, Swedish University of Agricultural Sciences, Almas Allé 8E, Box 7007, 750 07 Uppsala, Sweden; 3 Department of Arctic Biology, University Centre in Svalbard, UNIS, Box 156, 9171 Longyearbyen, Norway

**Keywords:** Citizen science data, Motivation, Observer behaviour, Sampling bias

## Abstract

Opportunistic reporting of species observations to online platforms provide one of the most extensive sources of information about the distribution and status of organisms in the wild. The lack of a clear sampling design, and changes in reporting over time, leads to challenges when analysing these data for temporal change in organisms. To better understand temporal changes in reporting, we use records submitted to an online platform in Sweden (Artportalen), currently containing 80 million records. Focussing on five taxonomic groups, fungi, plants, beetles, butterflies and birds, we decompose change in reporting into long-term and seasonal trends, and effects of weekdays, holidays and weather variables. The large surge in number of records since the launch of the, initially taxa-specific, portals is accompanied by non-trivial long-term and seasonal changes that differ between the taxonomic groups and are likely due to changes in, and differences between, the user communities and observer behaviour.

**Supplementary Information:**

The online version contains supplementary material available at 10.1007/s13280-021-01550-w.

## Introduction

Online portals through which volunteers submit reports of observations of wildlife gather large amounts of data worldwide. The majority of such species occurrence data are collected opportunistically with little or no underlying sampling design, but often with much higher temporal and spatial resolution compared to designed studies (Ruete et al. 2017). The wide availability and cost-effectiveness of opportunistic data, and the public engagement benefits they can provide, leads to stakeholder interest from governmental agencies, non-governmental organisations, universities and research institutes. For instance, governmental agency staff may use the data for infrastructure development planning or red-listing of species (Maes et al. 2015), and researchers may be interested in distributional range shifts (Prieto-Torres et al. 2020). 

The opportunistic nature of the data leads to large variability in reporting that is due to a largely unknown mix of variation that can be ascribed to observer behaviour (August et al. 2020) and variation due to ecological processes. This variation poses challenges when data are used for inference about populations, and also leads to lack of trust in inferences (Burgess et al. 2017). To gain a solid understanding of how variation in effort affects inference about ecological processes, and to be able to incorporate variation in effort in statistical models to correct for bias (Altwegg and Nichols 2019), we need to first understand the nature of variation in reporting. Previous studies have looked at how reporting varies spatially with anthropogenic variables such as road access (Mair and Ruete 2016; Zhang 2020) and human population density (van der Wal et al. 2015), and among observers (August et al. 2020). Few studies have investigated the details of temporal variation (but see Otegui et al. 2013; Zhang 2020). Reporting is, however, expected to vary in time due to variation in availability of organisms over seasons and years; with factors affecting observer behaviour and movements, including weekends (Żmihorski et al. 2012), holidays and weather; and with factors affecting observer reporting behaviour such as database reporting gaining in popularity (Amano et al. 2016), targeted efforts to increase reporting engagement (Sullivan et al. 2014), changes in reporting interfaces and functionality (August et al. 2015), and with changes in the community of active users. Temporal variation in effort arising from changes in observation and reporting behaviour need to be considered when making inferences about ecological processes in time, such as when estimating trends in occurrence or abundance, range shifts and trends in spatial distribution, and seasonal patterns (phenology) or their long-term trends. Studies using opportunistic citizen science data to estimate temporal trends have stressed the importance of correcting estimates for variation in effort, but results of attempts to validate estimated trends against trends derived from studies with stronger designs have been mixed (Snäll et al. 2011; van Strien et al. 2013; Kamp et al. 2016; Boersch-Supan et al. 2019). A better understanding of reporting patterns might eventually shed some light on when correction attempts may be successful, how to do them, and what might be done to the reporting system to increase the usefulness of data. For instance, knowledge of variation in reporting may be used to inform simulations manipulating specific mechanisms believed to cause bias and then checking the effects of those mechanisms on trend estimates (Isaac et al. 2014). Some methods to correct for observer bias also rely directly on an understanding of variables influencing variation in effort (Johnston et al. 2020).

Our aim in this study is to investigate broad temporal patterns in reporting of birds, butterflies, beetles, vascular plants and fungi to the Swedish Species Observation System (Artportalen; Shah and Coulson 2021). We examine patterns at a daily resolution to understand how reporting has changed during the period from 2000, when online reporting for birds was first launched, to 2018. Specifically, we decompose change in reporting into long-term and seasonal patterns, effects of weekdays and holidays, and simple weather variables, and compare these patterns among the taxonomic groups.

## Materials and Methods

### Data

#### Response variables

The Swedish Species Observation System (Artportalen; https://www.artportalen.se/) is a web portal and database to which the public can submit reports of species observations across taxa from plants to animals, covering all multicellular taxa, currently holding 80 million records. The main reporting user group is the general public, but the system is also integrated with the authorities reporting of survey-based biodiversity data.

The data are largely ‘presence-only’, i.e., records of what has been observed, not of what has not been observed (Gelfand and Shirota 2019; an option for checklist reporting has, however, recently been added to the system). There is no requirement on adhering to any specific sampling design: observers choose which species to report, where to observe them, when, and what amount of time and effort to devote. These aspects of the data imply that the vast majority of data can be called ‘opportunistic’. However, some data from systematic surveys, with varying degrees of standardisation, are also submitted.

Since the launch of the web-based reporting system in 2000 several substantial changes were made, including adding platforms for different organism groups, and merging separate platforms into one single unified platform (Table 1). Here we make use of Artportalen’s 20-year history with known changes, many well-recorded species groups with high temporal density of data, and opportunistic nature of a majority of observations to investigate temporal patterns in recording.

**Table 1:** Major changes to what is today the Swedish Species Observation System, Artportalen, artportalen.se

Year		Description of system change
2000	June	Launch of the web platform for birds (Aves) ‘Artportalen’
2003	Autumn	Launch of the web platform for plants (Tracheophyta)
2003	Autumn	Launch of the web platform for butterflies and moths (Lepidoptera)
2003	Winter	Launch of the web platform for fungi
2006	Spring	New web platform for birds (replacing the former platform)
2006	Autumn	New web platform for invertebrates (including insects and spiders; replacing the former platform for butterflies and moths
2007	Spring	Launch of the web platform for vertebrates (including all other vertebrates except birds, bats, and fish)
2007	Spring	Launch of the web platform for fish
2007	Summer	New web platform non-animal groups (plants, fungi, mosses, lichens and algae)
2007	Autumn	Launch of the web platform for marine invertebrates
2013	May	New web platform Artportalen 2 (merge of several of the former separate platforms: plants, fungi, mosses, lichens, algae, vertebrates other than birds)
2014	May	Inclusion of invertebrates in Artportalen 2
2015	April	Inclusion of birds in Artportalen 2

The data consist of records of observations of at least one individual of a single species from a single location by an observer at some point in time. There may be multiple records of single and/or multiple species at the same place and time by the same observer. In some cases there are repeat records of the same individual(s) of a species from multiple observers. Using the Swedish LifeWatch Analysis Portal (Leidenberger et al. 2016), we extracted all records between 2000 and 2018 of species from five selected species groups that had a large number of records compared to most other groups: birds (45 million records), butterflies (0.9 million), beetles (order *Coleoptera*; 0.5 million), vascular plants (division *Tracheophyta*; 4 million), and fungi (division *Basidiomycota*; 1.2 million). We removed observations with insufficient temporal resolution (recording time exceeding 1 day) and with uncertain species identification.

We computed two sets of response variables for each species at a daily resolution. The first set aims to explore how the number of records has changed over time and consists of three response variables: the total number of records each day, the total number of unique observers each day, and the number of records per observer each day. The total number of records serves as a measure of how the amount of data collected has changed over time. The total number of observers and the number of records per observer, provides additional information about how this change has come about.

The second set of response variables aims to explore whether there is variation over time in the locations from which observations come. We used locations of ‘species lists’, defined as a set of observations of different species made by the same observer on the same day and at the same locality (same geographical coordinates) (Szabo et al. 2010), instead of locations of individual records to avoid repeating the spatial variable over multiple species records reported by an observer from a single location. From the locations of all lists within a day, we computed the mean of two spatial response variables: latitude and human population density. Via latitude, we investigated whether there may be temporary or permanent shifts in volume of observations towards more southern or northern locations over time, i.e. different parts of the country that differ in reporting effort. Using human population density we investigated how the proportion of records from highly populated versus more sparsely populated areas varied over time. We aggregated human population density at a 10 × 10 km resolution by summing population sizes from raster data at higher resolution (SCB Statistics Sweden; https://www.scb.se/vara-tjanster/oppna-data/oppna-geodata/statistik-pa-rutor/). We subsequently log(*x* + 1) transformed human population density at the location of species lists before computing the spatial average to not give exessive weight to lists from the larger cities (large skewness of the distribution of population sizes at the arithmetic scale; Mair and Ruete 2016). Human population distribution has not gone through any major changes during the study period, justifying the use of a snapshot map of population size.

#### Weather

We extracted mean daily temperature and total precipitation across 0.25° grid squares covering the whole of Sweden (Cornes et al. 2018). Temperature has a strong seasonal and spatial pattern, and to reduce confounding with a general seasonal pattern in reporting we computed a detrended temperature variable. Specifically, we fitted a cyclic smooth seasonal curve to the time series of daily temperature from each grid square. The daily residuals from these curves were averaged across all grid squares to compute a daily temperature deviation index, which was used in analyses of reporting data. For precipitation, seasonal patterns are weaker, and we therefore used daily average precipitation across all grid points without detrending. As these variables are averages across the country, they will tend to reflect large-scale weather events rather than local weather.

### Models

We analysed temporal patterns in the data using generalised additive models (GAMs). We denote the response variable *y*_*t*_, where *t* is the number of days since January 1 in year 2000. The basic structure of the model was:

$$y_{t} \,\sim \,s\left( t \right)\, + \,s\left( {doy} \right)\, + \,dow\, + \,holiday\, + \,c_{temp} \left( {doy} \right) \, \cdot \, temp\, + \,c_{rain} \left( {doy} \right) \, \cdot \, rain$$

Here, *s(t)* is a smooth function representing slow, long-term, changes in reporting over time; *s(doy)* is a smooth function of the day of year (*doy*), representing seasonal patterns; *dow* is a fixed factor with a level for each day of the week; *holiday* is a fixed factor with 15 levels (14 public holidays over all years, plus an extra level for no holiday); *c*_*temp*_*(doy)* and *c*_*rain*_*(doy)* are coefficients for the effects of temperature deviation and rainfall, which are allowed to vary over the seasons.

We modelled the long-term trend *s*(*t*) using a thin plate spline with 10 degrees of freedom, and the seasonal smooth *s*(*doy*) using a thin plate spline with 40 degrees of freedom. For the seasonal smooth, we did not use a cyclic spline (i.e. with matching levels at the start and end of a year), despite that it may seem an obvious choice, because we expected higher reporting in the beginning than in the end of a year with a resulting possible discontinuity in the response variables with the change from one year to the next. This is mainly relevant for birds, for which many observers tally the number of species seen in a calendar year (Hui 2013), but we kept the same structure for all species groups. For the coefficient functions *c*_*temp*_(*doy*) and *c*_*rain*_(*doy*) we used cyclic cubic regression splines with 40 degrees of freedom. To investigate seasonal changes in the effect of weekends we allowed the seasonal pattern s(*doy*) to differ between weekends and weekdays.

In a separate model fit, we added an interaction term between seasonal variation and long-term trends to investigate whether there were indications of a difference in the long-term trends among different parts of the season. For this, we used a tensor product interaction based on a thin plate spline with 10 degrees of freedom for the long-term component and 6 degrees of freedom for the seasonal component. We held the degrees of freedom for the seasonal component low in the interaction to reduce the risk of identifiability issues between the long-term and seasonal components. In these separate model fits we also added an interaction term between long-term trends and weekend effects.

When the response was the number of records or the number of observers we used a log link and a negative binomial distribution with a quadratic variance-mean scaling. To model the average number of records per observer, we used the number of records subtracted by the number of observers as the response under a negative binomial distribution with the (log-transformed) number of observers as an offset. The number of observers was subtracted in the response variable because the number of records is always at least as large as the number of observers. This response will therefore always be positive and can attain the value zero, as assumed by the negative binomial model. For the spatial response variables average latitude and average log human population density we used a Gaussian response distribution. Since these responses were computed as averages, their variance will differ depending on the number of data points underlying the average. We therefore used the number of species lists as weights for the spatial response variables.

We tried to account for residual autocorrelation by first fitting the above model assuming no autocorrelation. From that fit we computed the empirical lag 1 autocorrelation of the residuals, which was then set as a fixed value in a final (second) fit of the model. Data for 2018 were withheld from model fitting and used to visually assess the predictive performance of the model. We fitted models using the functions *gam* and *bam* in the R package *mgcv* (Wood 2006).

## Results

**Fig. 1:**
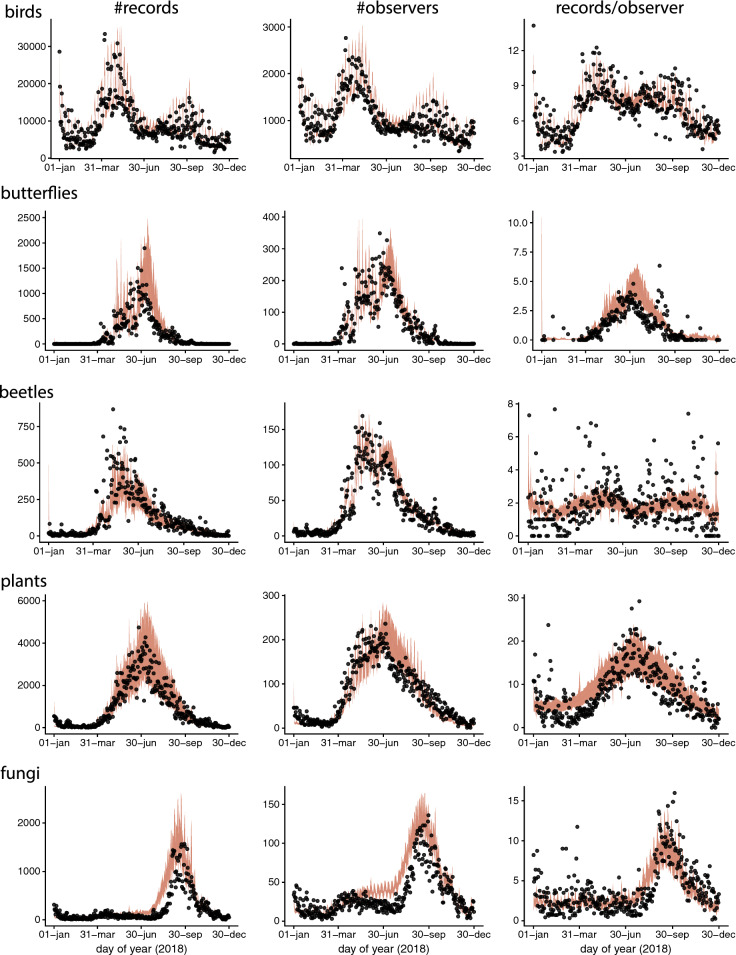
Daily number of records (left panels), observers (middle) and records per observer (right panels) for each species group with predicted mean (orange curve) vs observed (points) number of daily records for 2018 (right column). Predictions for 2018 are based on models fitted to data from year 2000 to 2017

Raw data for the per day, number of records, observers and records per observer for all species groups in 2018 are shown in Fig. 1. Forecasts for those numbers captured the main seasonal patterns for most groups, but overestimated the number of records of fungi and butterflies during the peak period in 2018.

### Long-term trends

**Fig. 2:**
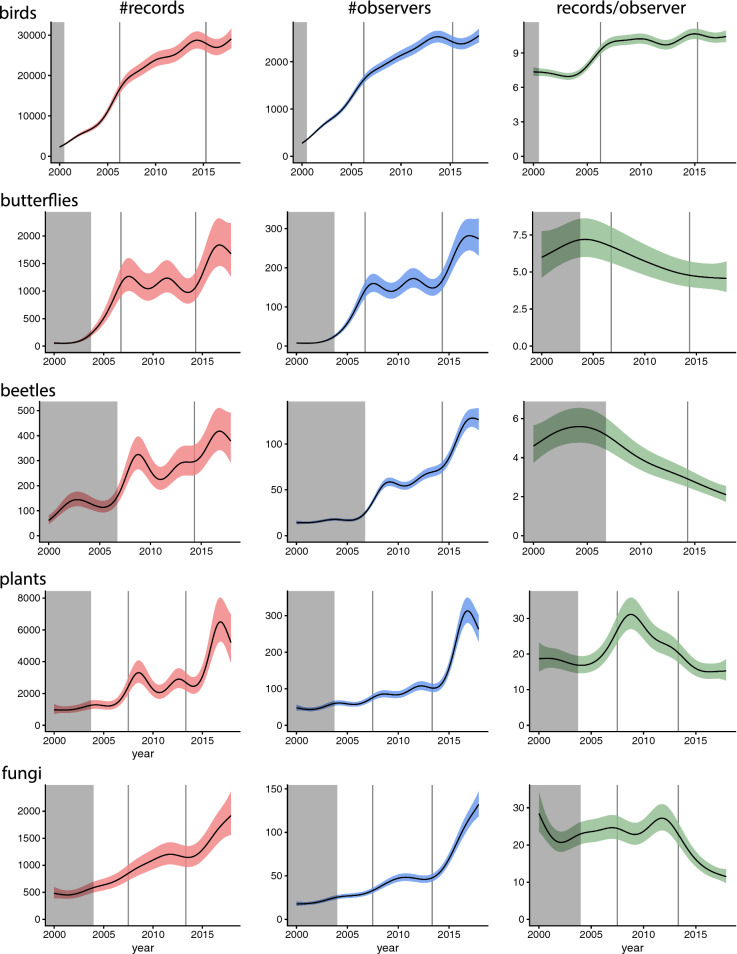
Estimated long-term trends in the daily number of records (left panels), observers (middle) and records per observer (right panels) for each species group. Trends are evaluated as predicted values for the peak season (estimated from the seasonal smooths of number of records). Shaded regions around trends refer to 95% confidence intervals. The shaded grey areas indicate the time period before the online platform was launched and hence constitute backlogs only. Vertical lines indicate times of major changes in the reporting system (see Table 1)

The number of records and the number of observers increased approximately three to fourfold (beetles, vascular plants, fungi) or more (birds, butterflies) over the study period (Fig. 2). Long-term patterns for the number of observers were qualitatively similar to the patterns for the number of records for most species groups (Fig. 2). Despite this, there were clear differences in patterns for the number of records per observer—the fraction of the previous two responses.

The number of birds recorded per observer increased over the study period (Fig. 2). This increase happened mainly in the first years after the launch of the online portal. For the other groups, the number of records per observer decreased during the later part of the study period. Thus, the increase in the total number of records for these groups was due to an increase in number of observers, and happened despite observers on average submitting fewer records.

**Fig. 3:**
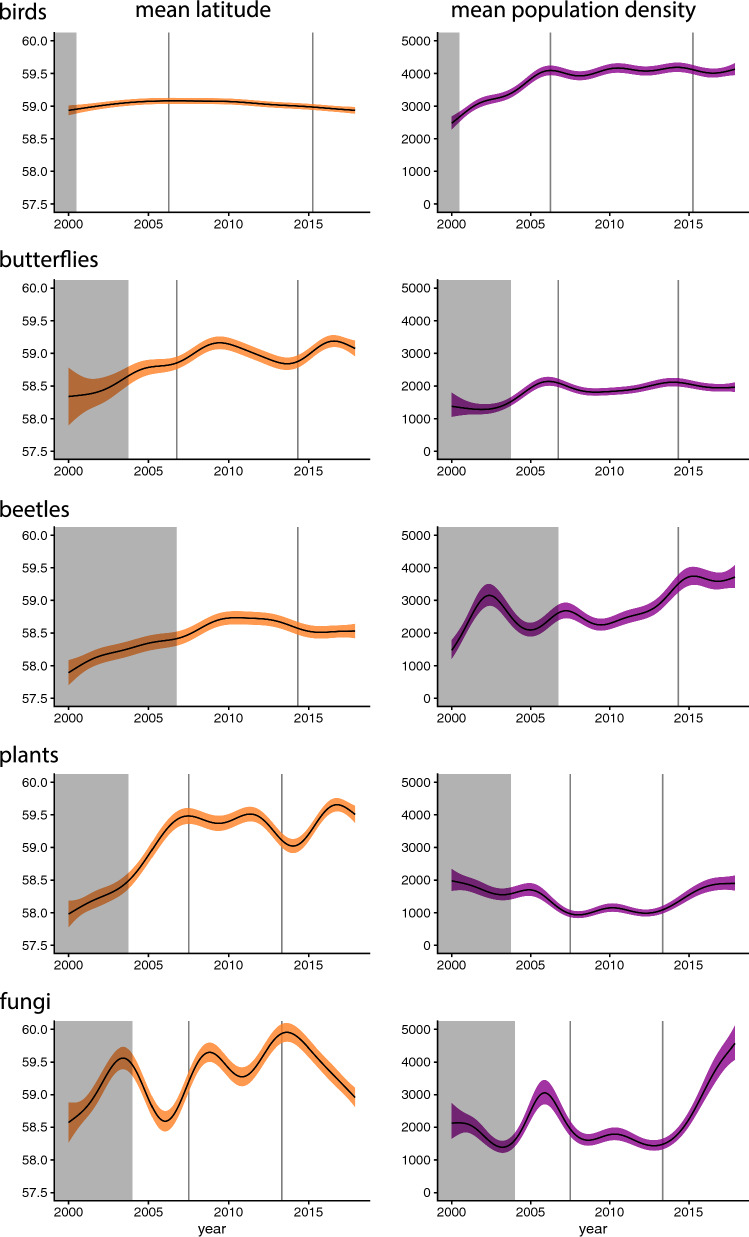
Estimated long-term trends in the average latitude (left panels) and average human population density (computed as the predicted geometric mean of population density + 1) at list locations (right panels) for all species groups. Trends are evaluated as predicted values for the peak season (estimated from the seasonal smooth of number of records). Shaded regions around trends refer to 95% confidence intervals. The shaded grey areas indicate the time period before the online platform was launched and hence constitute backlogs only. Vertical lines indicate times of major changes in the reporting system (see Table 1)

The average latitude of locations of species lists was largely stable across years for birds (Fig. 3), but lists tended to come from more densely populated areas during the latter part of the study period, due mainly to an increase in the first few years (Fig. 3). There was an increase over time in the mean latitude of lists for butterflies and vascular plants, and the number of lists from more densely populated areas for fungi. Other patterns for latitude and population size were more complex or less evident (Fig. 3)

A substantial change was made to the reporting systems between 2013 and 2015 when the taxa-specific platforms were merged, in stages, into a joint system (Table 1). Following this change the number of observers and records generally increased, except perhaps for birds, while records per observer decreased for beetles, vascular plants and fungi (Fig. 2). For these groups, lists came from on average more densely populated areas after the merger.

### Within-year patterns

#### Seasonal

**Fig. 4:**
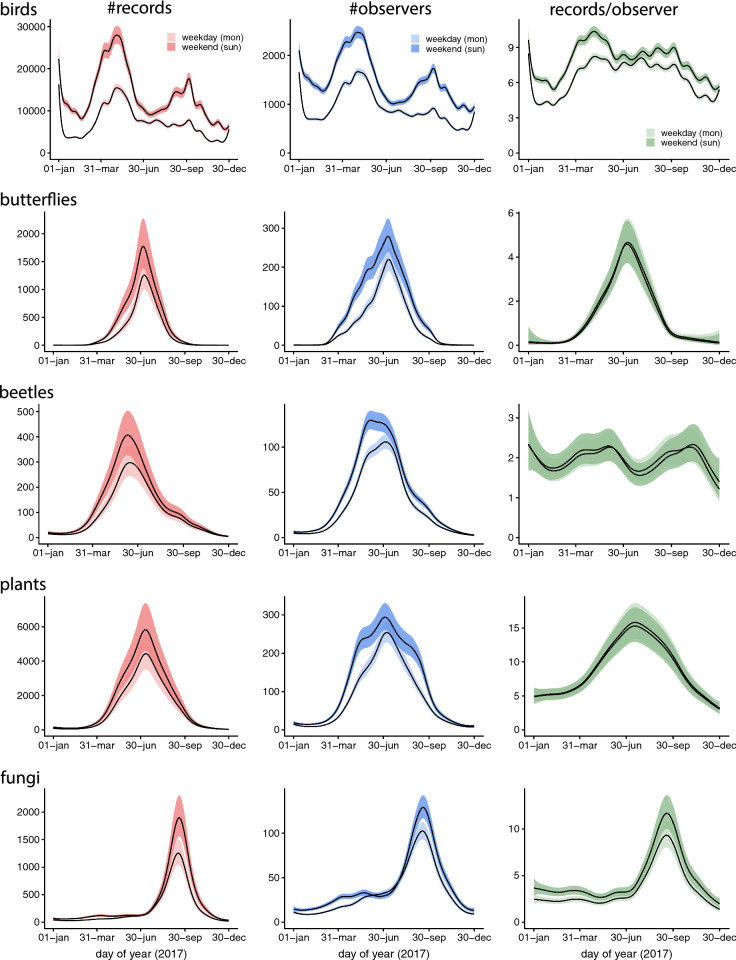
Estimated seasonal trends in the number of records (left panels), observers (middle) and records per observer (right panels) for each species group and for both weekdays and weekends. Trends are evaluated as predicted values for year 2017. Shaded regions around trends refer to 95% confidence intervals

Each species group revealed a somewhat different pattern of seasonality: multimodality in bird reports (records, observers, records per observer); mostly peaked curves for reports of other groups, but at different times of the year; with a plateaued curve for beetle observers; vascular plant observers showing abrupt transitions between small and large numbers; and beetle records per observer with multimodality (Fig. 4).

The number of records and the number of observers for birds showed a complex pattern with a sharp peak immediately after New Year, as well as broader peaks during the migration periods in notably spring but also autumn. For all groups the average number of records per observer tended to peak at approximately the same time of the year as the total number of records, except for beetles for which there was no clear peak (Fig. 4).

**Fig. 5:**
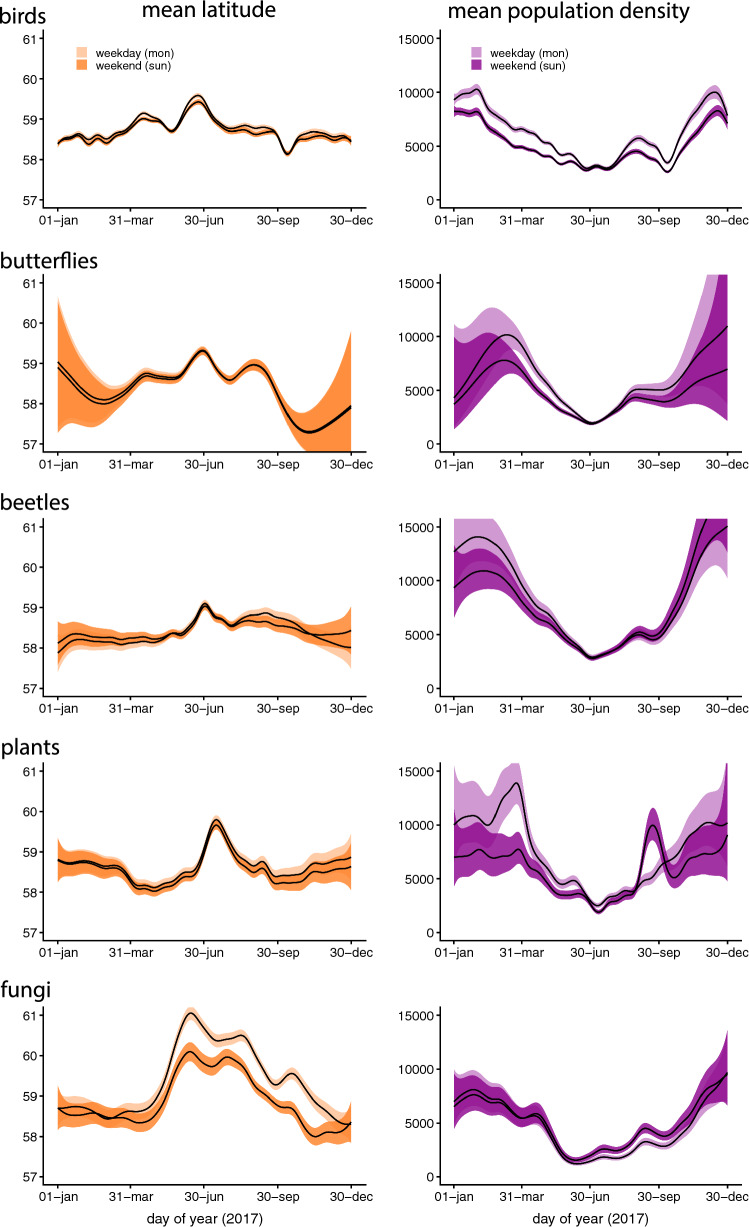
Estimated seasonal trends in the average latitude (left panels) and average human population density (computed as the predicted geometric mean of population density + 1) at list locations (right panels) for all species groups and for both weekdays and weekends. Trends are evaluated as predicted values for year 2017. Shaded regions around trends refer to 95% confidence intervals

Most groups showed strong seasonal patterns in the average location of lists (Fig. 5). Generally, lists on average came from less densely populated and more northerly located areas during summer than during winter. Here too, bird reports had the most complex seasonal pattern (Fig. 5).

#### Weekends

The number of records and number of observers tended to be higher on weekends for all species groups (Fig. 4). This effect was most pronounced for birds, with the number of records increasing by a factor of 2 or more during some periods of the year, and for beetles. For birds, the difference between weekends and weekdays was larger during all seasons outside the summer holiday period (late June and July), but we found no clear evidence for seasonally changing strength of weekend effects for the other species groups. For birds, also the number of records per observer was higher on weekends and holidays than during weekdays. Weekend effects on list locations were mainly small, but bird lists in spring and autumn tended to come from less populated areas, and lists of fungi from lower latitudes during weekends (Fig. 5).

#### Holidays

**Fig. 6:**
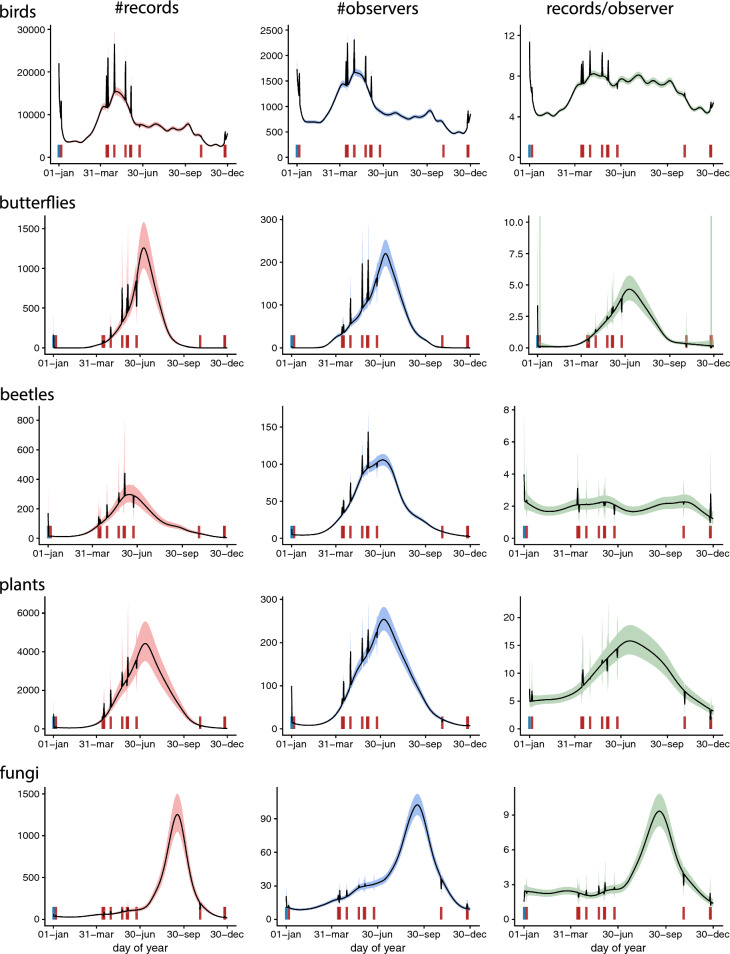
Estimated effects of holidays on the number of records (left panels), observers (middle) and records per observer (right panels) for each species group. Effects are overlaid on the seasonal curves and are evaluated for year 2017. Shaded regions around lines refer to 95% confidence intervals. Bars at the bottom of plots mark public holidays occurring on weekdays (red) and weekends (blue)

Holiday effects were mostly positive and strongest for birds with almost double the expected number of records for some public holidays in spring (Fig. 6). New Year’s Day had the strongest holiday effect, and often had the most bird reports among all days of the year, despite the number of bird species present in Sweden being considerably lower in winter. There were potential holiday effects also for beetles, butterflies and vascular plants, but these signals were weaker and less certain. There were no clear holiday effects for fungi, but there are few public holidays in autumn in Sweden when most fungi are reported. We found no strong holiday effects on average list locations, although there was some indication of bird reports coming from less populated areas on some spring holidays (Fig. S1).

#### Weather

All groups showed evidence for effects of daily temperature deviation and amount of rain on the number of records and observers, and many of those effects varied with season (Fig. S2). Effects of temperature deviation were mainly positive, while effects of rain were negative, so that more records were on average submitted for days with good weather (warmer than average for the time of the year, low precipitation) than on days with less good weather. The strength of the weather effects differed between the species groups and were found strongest for butterflies.

Generally, weather effects tended to be strongest in spring and weakest during summer. Overall, patterns were similar for average number of records per observer, but weaker and more uncertain, and for butterflies the negative effect of rain was constant throughout the year (Fig. S2). Effects of weather on the spatial responses were mostly uncertain, and effect sizes relatively small (Fig. S3).

#### Interactions between long-term trends and within-year patterns

The long-term trends in number of records were approximately proportional across seasons (Fig. S4). There were some deviations from this pattern, but these were generally small, or occurred for parts of the season with few records (mainly butterflies in winter). We found no strong indications of weekend effects changing in strength over years for any of the groups.

## Discussion

Our analyses illustrate broad temporal patterns in reporting observations for five species groups across more than a decade of database expansion. Below we discuss potential reasons for, mechanisms behind, and implications of, the long-term and within-year patterns.

### Long-term trends

The total number of records has increased by several orders of magnitude over the last two decades, a feature shared with many other online reporting systems (e.g. eBird, iNaturalist, ornitho.de, waarneming.nl; Amano et al. 2016). Strong increases often followed the launch of the, initially separate, taxa-specific online reporting systems. As found for other systems (Pocock et al. 2015; Arts et al. 2020) the number of records seemed to be influenced by infrastructural investment and communication around those, which were followed by strong increases for particularly plants and fungi. In order to get many records into the system from times past, substantial efforts were made to persuade collectors to digitise their records and report them (H. Ljungberg, pers. comm). This may have led to a greater average number of records per observer for species groups such as beetles and butterflies before Artportalen was launched for them. The long-term trends were qualitatively similar for the number of observers for most species groups. This, together with the general decrease or weaker increase in number of records per observer, suggests that the total increase of records is mainly driven by the increase in number of active observers contributing (see also Zhang 2020).

We do not expect any major changes in the overall abundance, availability or locations of organisms at the level of the species groups across the years covered in this study. Under this assumption, changes in the number of records per observer, in the latitudinal centroid, and in the mean human population density at report locations over years all suggest that observer/reporting behaviour is also changing in more subtle ways. We suggest that these changes may be due to (1) within-observer changes, (2) changes in the user community, and (3) changes in the reporting system, all relating to observers’ preferences. Users are often heterogeneous with variation in observation and reporting behaviour that may be described by e.g. their temporal and spatial activity pattern, taxonomic scope and amount of observations reported (Boakes et al. 2016; August et al. 2020). Such observer preferences and behaviours (engagement profiles) can change over time, through changes in the user community or within-observer changes (Rotman et al. 2012).

For birds, the increase in number of records per observer happened mainly in the first few years after the launch of the online portal. Making it easy to participate, for an already active community, likely contributed to within-observer changes and changes in the observer community with increased reporting of inventories (Wood et al. 2011). In contrast, for butterflies and beetles, the number of records submitted per observer have gradually decreased since the launch of the reporting systems. Additional analyses (Appendix S1) suggest that for beetles, part of this decrease may reflect a change in observer community. Participants that entered the system early on may have had a good knowledge of beetle species and therefore been able to identify and report many species. New users in later years reported fewer species on average (Fig S5), which could stem from a lower level of knowledge of this species group. Decreases in records per observer are found in later years also for plants and fungi, and largely coincide with increased reporting from more densely populated areas (Lopez et al. 2020). These declines cover the period when the taxa-specific platforms were merged, but appear mainly linked to declines in reporting by early users (Fig. S5). Long-term changes in average latitude for species lists may reflect changes in the user community (e.g. recruitment of new users in the north) affecting the spatial distribution of reports. One could imagine that on a longer time scale in the future, range shifts in species distributions could also start to drive changes in observer behaviour.

### Within-year patterns

#### Seasonal patterns

For all species groups, the availability of organisms varies over the season, which was reflected in the seasonal patterns of the total number of records, the number of observers, and the number of records per observer. These patterns mainly peaked at the time of year when organism activity and detectability is highest—with one summer peak for butterflies, beetles and plants, one autumn peak for fungi, and spring and autumn peaks for birds. Activity and detectability may both give rise to similar patterns in the number of records, number of observers, as well as in spatial variation and effects of weather. There are also likely reinforcements in these relationships, e.g. the arrival of migratory birds (Greenwood 2007) and emergence of insects and plants in spring (Daru et al. 2018) attract more observers and lead to increased effort and reporting (Lawrence 2009). These effects may be exacerbated during favourable weather conditions that affect activity patterns of non-sessile organisms (birds, butterflies, beetles) which impacts detectability, which in turn may increase observer effort.

How details of observers’ behaviours may impact seasonal patterns we illustrate with three examples. Bird reports showed some detailed seasonal patterns with a substantial additional peak during the first few days of the New Year. Many bird watchers, in Sweden and elsewhere, keep annual species lists and compete for observing as many species as possible starting each year on 1 January (Hui 2013). The spike after New Year suggests that competition can contribute to the motivation to report more of the species that have been observed (more complete reporting, including common species that may go unreported during other field excursions). The details of observers’ behaviours may therefore impact seasonal patterns, and these may differ greatly among taxa. For beetles, the number of records submitted per observer was fairly constant over the season. One potential reason for this is that observations of beetles in winter is often done by sifting soil for hibernating adults, which can lead to long species lists (H. Ljungberg, pers. comm). And increased reporting from birders gathering at popular migratory hotspots along the coast of southern Sweden likely explain the decrease in latitude of bird lists during the autumn migration period.

#### Weekends/holidays

In contrast to seasonal patterns, effects of weekends and holidays are expected to be nearly exclusively due to observer behaviour. Similar to other studies (Żmihorski et al. 2012; Otegui et al. 2013; Zhang 2020) the total number of records and observers were higher during weekends for most groups, with the strongest effects for birds (Courter et al. 2013). Birds are also the only group for which there was a clear holiday effect (see also Surmacki 2005). More observations made during weekends and holidays is expected when many amateur naturalists of working age are contributors of data. In agreement with this, bird reports also tended to come from less densely populated areas in weekends, presumably due to more time available for travelling to targeted birding locations.

The difference in strength of the weekend effect between organism groups may be partly due to sample size as the number of daily records was ≥ 10 times larger for birds than for the other groups. But the difference may also be due to intrinsic differences in observer behaviour or the observer community. For example, if a larger proportion of reports come from retirees, activity is likely more evenly spread independently of weekday. Similarly, a large portion of records from professional surveys carried out mainly during the working week could reduce or mask weekend effects of amateur observers.

#### Interactions between seasonal and annual patterns

Overall there were no strong indications for clear changes in seasonal or weekend patterns over years. Despite the variation in reporting patterns over years discussed above, there therefore seems to be some degree of stability in reporting patterns across large increases in total reporting and changes to the underlying reporting system.

### Implications for inference

Multiple factors contribute to the motivation for reporting species observations to public online databases (Hobbs and White 2012; Maund et al. 2020). Motivation in turn affects observer behaviour, in terms of the willingness to report observations and in the effort spent on observing species. When opportunistic data are used with the aim of studying temporal patterns in taxa, shifts in observer motivation over time can be problematic for inferences because changes in observer behaviour may be mistaken for, or confused with, changes in taxa. Temporal changes in detectability, e.g. due to changes in species activity, changes in survey methods, or observer experience, are well-known issues when estimating temporal changes from data collected under targeted and designed surveys (Barker and Sauer 1992). Similar issues occur in opportunistic data, but changes in motivation add another layer of complexity that exacerbates the problem of drawing valid inference from data.

Some of the key temporal patterns in reporting suggested by our analyses were: (I) large increases in reports and observers across all species groups; (II) long-term changes in records per observer and in the spatial locations of reports, differing among the groups, and likely arising from changes in reporting behaviour and changes in user communities following developments in the reporting platforms; (III) seasonal patterns in records per observer and spatial locations of reports, likely partly resulting from cultural behaviour among observers within the different taxonomic groups.

A parallel increase in observers and records, with no or little change in observer behaviour, will often be easy to correct for in analyses of temporal change. For example, Isaac et al. (2014) evaluated different methods for estimating long-term trends in distribution under simple simulation scenarios and found that all methods that addressed observer changes performed reasonably well when the number of observers increased over time. Long-term changes in effort pose more severe challenges. In the simulations of Isaac et al. (2014), some methods, particularly occupancy models, also performed well when correcting for changes in effort via lengths of species lists (Szabo et al. 2010). In practice, some studies have found trends in distribution derived using these corrections to not satisfactorily agree with estimates from designed studies (Kamp et al. 2016), while others have found better agreement (van Strien et al. 2013). These methods therefore seem to be partly able to correct for variation in effort for inference about changes in distribution, but it is less clear in which situations inferences are ‘good enough’ or how spatio-temporal variation in effort affects estimates of other types of change (e.g. range shifts, trends in abundance, or trends in phenology).

Our seasonal curves in records per observers were distorted relative to expected phenology curves, and differently so among the species groups. We suggest that these distorted seasonal patterns could be more difficult to correct for using species lists length. Effectively, list length corrections rely on the assumption that recording effort would be same if list lengths were the same. In seasonal environments, this will not hold true over the course of a year, and so alternative, or refined, corrections may be required. A better understanding of how motivation and behaviour of observers changes over the season could potentially generate new ways of adjusting for changes in effort.

## Conclusions

To improve biological inference about temporal change from opportunistic data, we argue that several steps will be necessary. First, we need to better understand temporal change in observer behaviour and motivation. Our study provides an initial attempt at this, but future studies could dig deeper into the mechanisms behind changes in effort over time and compare them among platforms. Second, improved understanding of change in effort can be incorporated in detailed scenario simulations (Isaac et al. 2014) to guide improvements in analytical methods as well as platform infrastructure in order to increase information content in the data. Third, promising analytical methods should be evaluated against independent data from designed monitoring studies. These steps will likely need to be performed for each taxonomic group, but together may improve trust in conclusions reached from analyses of opportunistic data (Bayraktarov et al. 2019).

## Supplementary Information

Below is the link to the electronic supplementary material.

Electronic supplementary material 1 (PDF 804 kb)
