# Supplementary material for: Temporal trends in opportunistic citizen science reports across multiple taxa
Source: Ambio. 2021 Mar 29;51(1):183–98. doi: 10.1007/s13280-021-01550-w (PMC8651922; doi:10.1007/s13280-021-01550-w)
Supplement: Supplementary file 1 — Electronic supplementary material 1 (PDF 804 kb) [file 13280_2021_1550_MOESM1_ESM.pdf]

Ambio

Electronic Supplementary Material
This supplementary material has not been peer reviewed.

Title: **Temporal trends in opportunistic citizen science reports across multiple taxa**

Authors: Jonas Knape, Stephen Coulson, René van der Wal, Debora Arlt

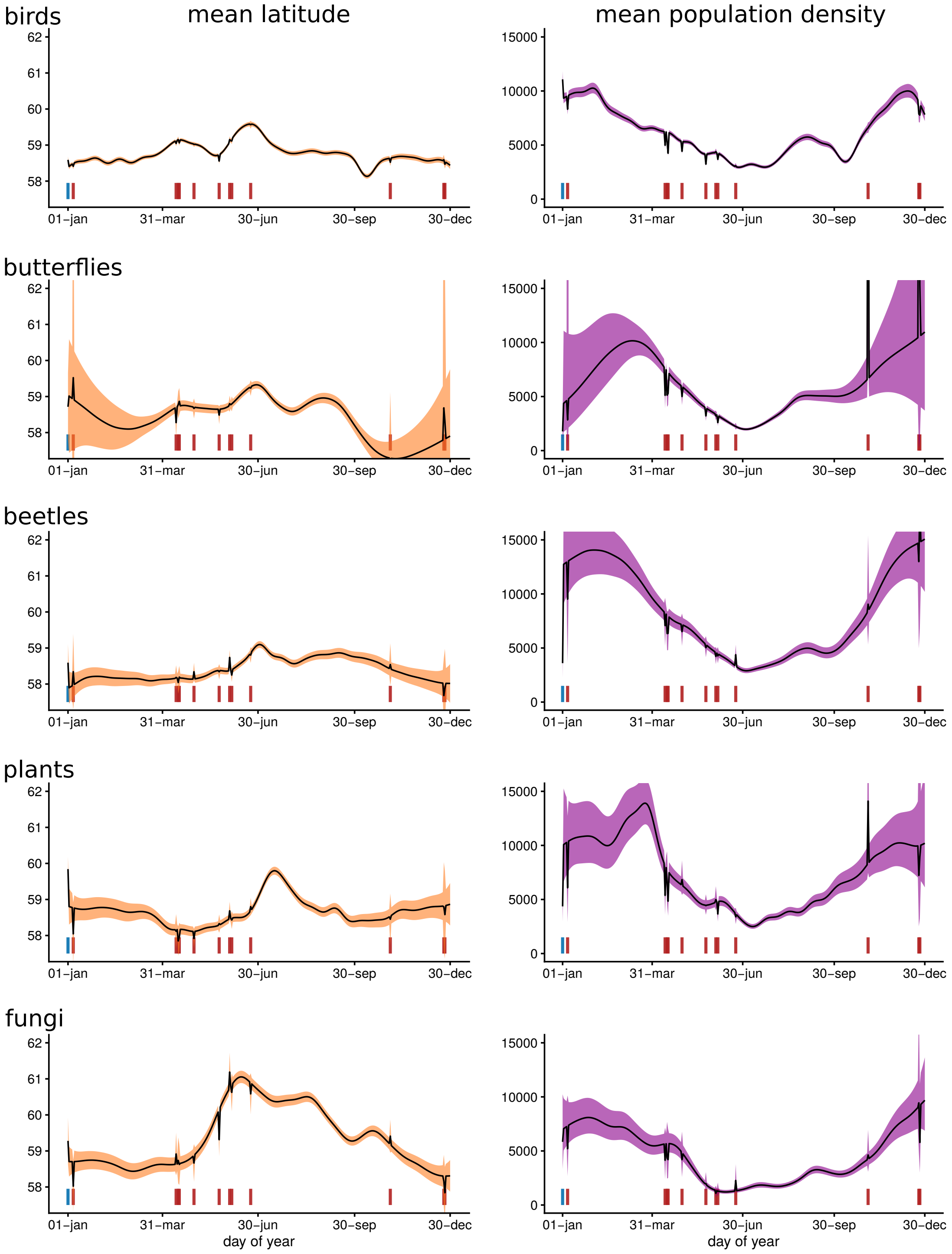

Figure S1. Estimated effects of holidays on the average latitude (left panels) and average (log(x+1) transformed) human population density of at list locations (right panels) for all species groups. Effects are overlaid on the seasonal curves and are evaluated for year 2017. Shaded regions around lines refer to 95% confidence intervals. Bars at the bottom of plots mark public holidays occurring on weekdays (red) and weekends (blue).

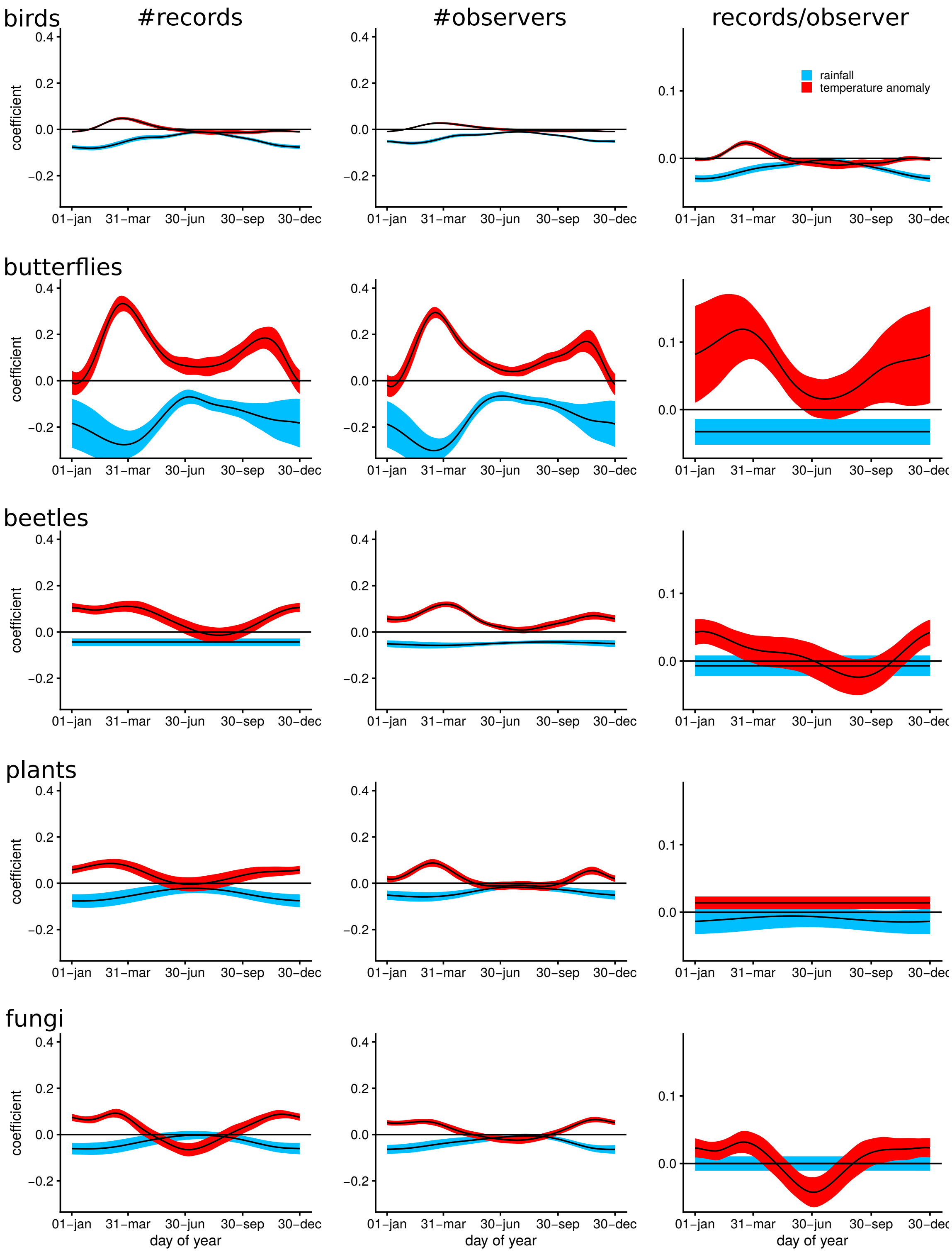

Figure S2. Regression coefficients (slopes) for the effect of daily temperature deviation and daily total precipitation on the on the number of records (left panels), observers (middle) and records per observer (right panels) for each species group. Shaded regions around lines refer to 95% confidence intervals. The coefficients are estimated as smooth cyclic curves varying over the season.

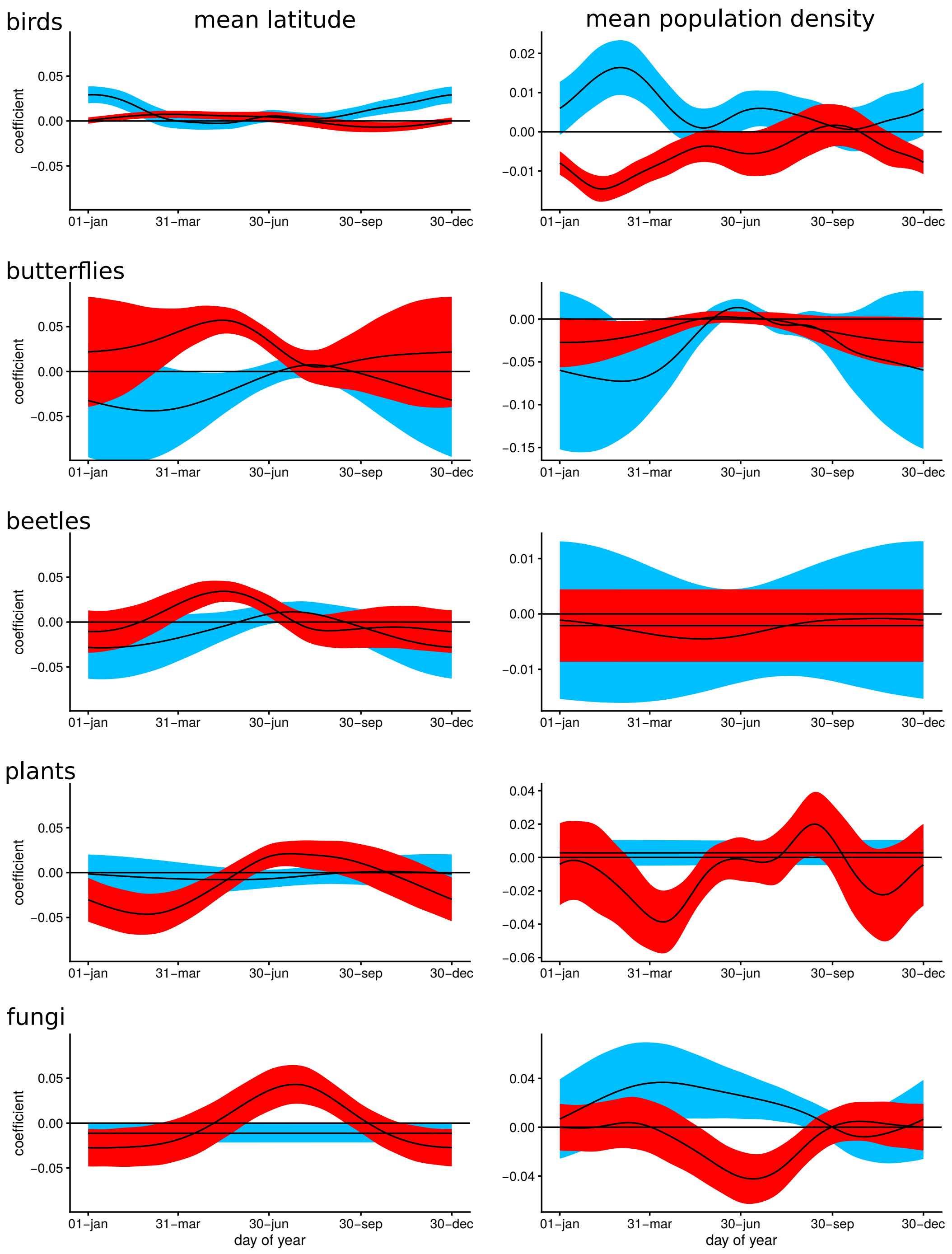

Figure S3. Regression coefficients (slopes) for the effect of daily temperature deviation and daily total precipitation on the average latitude (left panels) and average (log(x+1) transformed) population size at list locations (right panels) for all species groups. The coefficients are estimated as smooth cyclic curves varying over the season. Shaded regions around lines refer to 95% confidence intervals.

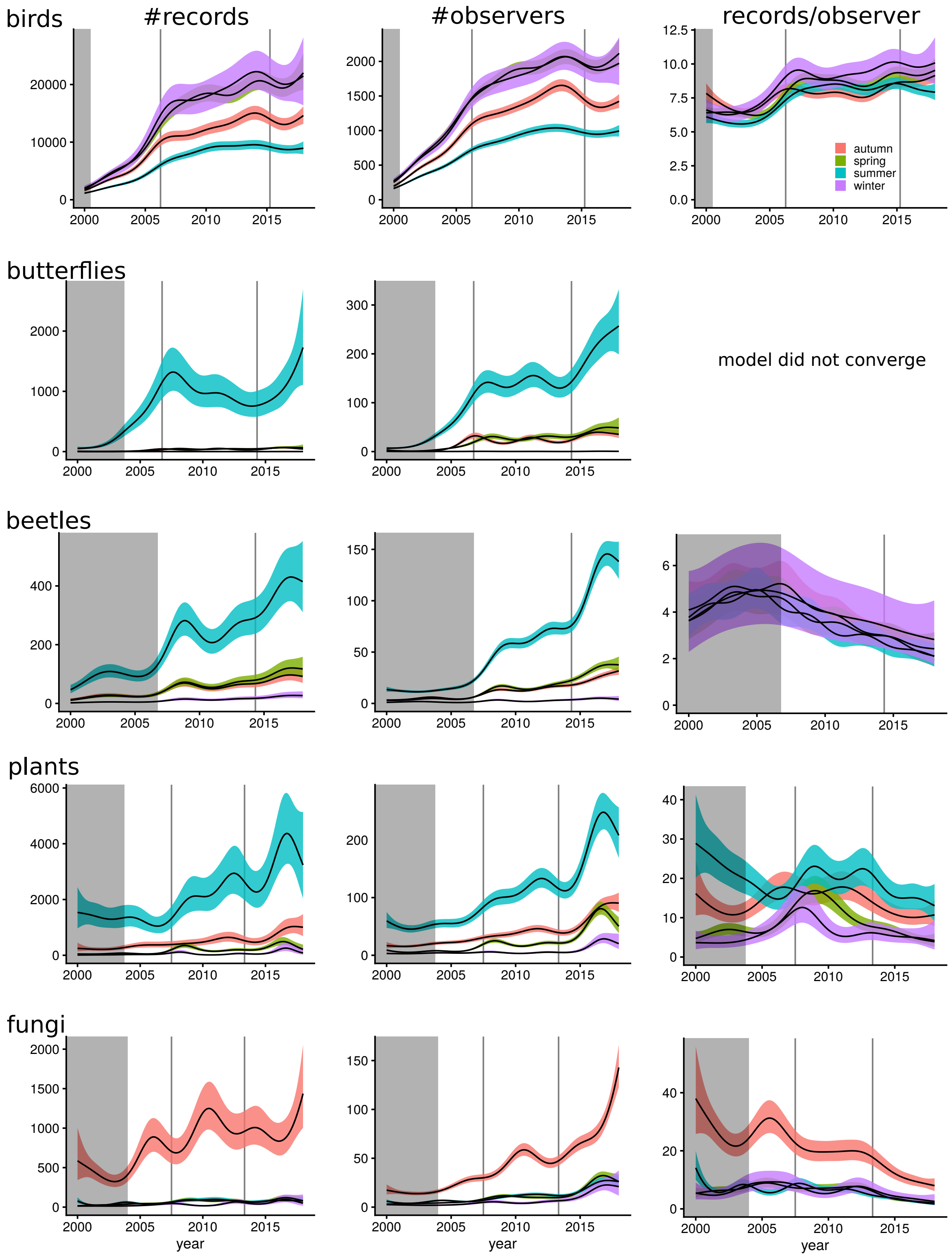

Figure S4. Estimated long-term trends in the daily number of records (left panels), observers (middle) and records per observer (right panels) for each species group when including interactions between seasonal and long-term change. Trends are evaluated as predicted values for winter, spring, summer and autumn. Shaded regions around trends refer to 95% confidence intervals. The shaded gray areas indicate the time period before the online platform was launched and hence constitute backlogs only. Vertical lines indicate times of major changes in the reporting system (see Table 1).

Appendix S1.

In a simplified analysis we explored the change in number of reports per observer in more detail. The motivation was to better understand whether changes were mainly due to within observer changes or to changes in the observer community. For this we used the same basic model structure as in the main paper, but excluded weather effects. In these analyses we fitted separate long-term trends depending on the time of entry of the observer into the system, defined as the date of the first record submitted by the observer. We used four categories with first records between 2000-2005, 2005-2010, 2010-2015, and 2015 or later. Note that a caveat with this is that we only considered the date of the first record, not the time the report was made. All other model components (seasonal trend, weekend and holiday effects) were for simplicity assumed to be the same across these groups.

We used three response variables in these analyses. First, we used the average number of records per list, where a list was defined as a collection of reports from the same observer on the same location and day. Second, we used the average number of lists per observer and day. Third, we considered the average number of records per observer, similarly to the analyses in the main text. A difference to the analyses in the main text is that we only counted records that constituted a species unique to the list they came from (i.e. multiple records of the same species from the same list, e.g. individuals of different sex or age, were only counted as one record). We focused on unique species in these analyses to try to better isolate effects of changes due to users with varying degrees of species identification expertise entering the system. For all of the above responses we used a negative binomial distribution for the numerator (e.g. number of lists) and included the denominator (e.g. number of observers) as an offset, as in the analyses of the number of records per observer in the main text.

Results of the above analyses are given in Fig. S5. They suggest that observers entering the system more recently tended to report fewer records. For birds and butterflies this seemed to mainly stem from more recent observers reporting fewer lists (i.e. from fewer locations), but with mostly approximately similar numbers of species per list as compared to observers entering the systems in the early 2000’s. For beetles, plants and fungi on the other hand, observers that entered the system more recently tended to report both fewer lists and fewer species per list. Overall declines in the number of records per observer for all groups except birds seemed to occur also in the group of observers entering the systems early.

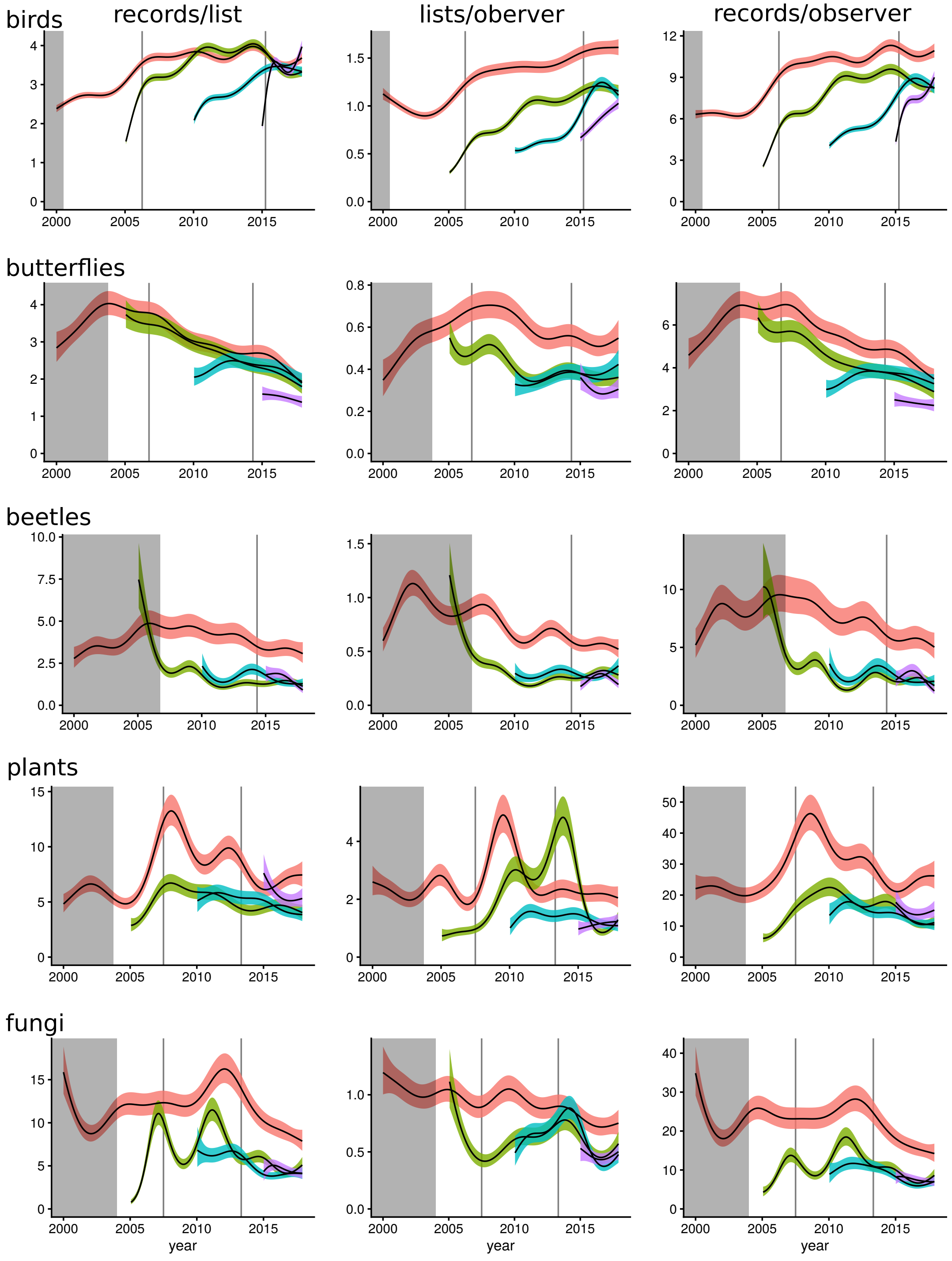

Figure S5. Estimated long-term trends in the number of records per list (left panels), lists per observer (middle) and records per observer (right panels) for each species group. Separate trend lines show trends for observers entering the system between 2000-2005, 2005-2010, 2010-2015, and after 2015. Trends are evaluated as predicted values for the peak season (estimated from the seasonal smooths of number of records). Shaded regions around trends refer to 95% confidence intervals. The shaded gray areas indicate the time period before the online platform was launched and hence constitute backlogs only. Vertical lines indicate times of major changes in the reporting system (see Table 1).
